# Characterization of microbiome and metabolite analyses in patients with metabolic associated fatty liver disease and type II diabetes mellitus

**DOI:** 10.1186/s12866-022-02526-w

**Published:** 2022-04-15

**Authors:** Qiuping Yang, Leisheng Zhang, Qian Li, Man Gu, Qiu Qu, Xinglong Yang, Qinghua Yi, Kunli Gu, Linli Kuang, Mei Hao, Jing Xu, Hongju Yang

**Affiliations:** 1 Division of Gastroenterology, The First Affiliated Hospital of Kunming Medical University, Kunming, 650031 China; 2 Key Laboratory of Molecular Diagnostics and Precision Medicine for Surgical Oncology in Gansu Province & NHC Key Laboratory of Diagnosis and Therapy of Gastrointestinal Tumor, Gansu Provincial Hospital, 204 Donggangxi Road, Chengguan District, Lanzhou City, 730000 Gansu Province China; 3 Key Laboratory of Radiation Technology and Biophysics, Hefei Institute of Physical Science, Chinese Academy of Sciences, 350 Shushanhu Road, Hefei, 230031 China; 4 Transfusion Medicine Research Department, Yunnan Kunming Blood Center, Kunming, 650011 China; 5 Kunming Guandu District People’s Hospital, Kunming, 650220 China

**Keywords:** Metabolic associated fatty liver disease (MAFLD), Type II diabetes mellitus (T2DM), Gut microbiota, Metabolome, Clinical diagnosis

## Abstract

**Background:**

State-of-the-art renewal has indicated the improvement of diagnostics of patients with metabolic associated fatty liver disease (MAFLD) and/or type II diabetes mellitus (T2DM) by dissecting the clinical characteristics as well as genomic analysis. However, the deficiency of the characterization of microbial and metabolite signatures largely impedes the symptomatic treatment.

**Methods:**

For the purpose, we retrospectively analyzed the clinical data of 20 patients with MAFLD (short for “M”), 20 cases with MAFLD and T2DM (short for “MD”), together with 19 healthy donors (short for “Ctr”). Microbial and metabolite analyses were further conducted to explore the similarities and differences among the aforementioned populations based on feces and blood samples, respectively.

**Results:**

Compared with those in the Ctr group, patients with M or MD revealed multifaceted similarities (e.g., Age, ALP, LDL, BUN) and distinctions in clinical indicators of liver (e.g., BMI, ALT, PCHE, CAP). With the aid of microbial and metabolite analyses as well as bioinformatic analyses, we found that the characteristics of gut microbiota (e.g., abundance, hierarchical clustering, cladogram, species) and lipid metabolism (e.g., metabolite, correlation coefficient and scatter plot) were distinct among the indicated groups.

**Conclusions:**

The patients with MD revealed multifaceted similarities and distinctions in characteristics of microbiome and metabolites with those in the M and HD groups, and in particular, the significantly expressed microbes (e.g., Elusimicrobiota, Berkelbacteria, Cyanobacteria, Peregrinibacteria) and lipid metabolites (e.g., Lipid-Q-P-0765, Lipid-Q-P-0216, Lipid-Q-P-0034, Lipid-Q-P-0800), which would collectively benefit the clinical diagnosis of MAFLD and T2DM.

**Supplementary Information:**

The online version contains supplementary material available at 10.1186/s12866-022-02526-w.

## Background

Metabolic associated fatty liver disease (MAFLD) also known as non-alcoholic fatty liver disease (NAFLD), including nonalcoholic steatohepatitis (NASH) and nonalcoholic fatty liver (NAFL), has been recognized as the leading cause of chronic liver disorder worldwide and the burgeoning public health issue [1–3]. Definitely, MAFLD is a spectrum of liver disorders with metabolic dysfunction and the accompanied clinical presence of steatosis in over 5% of hepatocytes, which commonly result in poor hepatic manifestation and prognosis such as cirrhosis, fibrosis and hepatocellular carcinoma (HCC) [2, 4, 5].

Longitudinal studies have suggested the continuous improvement in the diagnostics and management of MAFLD (e.g., liver biopsy, clinical indicators, therapeutic intervention), yet the patients still endure the well-known challenges such as long-suffering relapse, metabolic syndrome and immunological rejection largely attribute to the deficiency of parameter spectrum for dissecting the pathogenesis including gut microbiota and lipid metabolism [6, 7]. In particular, the design of clinical trials and the development of pharmacotherapies are far from satisfaction largely due to the current limitations in understanding the heterogeneity of MAFLD patients with multiple complications including essential hypertension (EH) and T2DM inaccuracies as well as the necessity of reappraising the terminology and definitions of the nomenclature [8–10].

Notably, state-of-the-art renewal has revealed diseases linked to dyslipidemia including atherosclerosis, obesity and MAFLD, which are associated with variations in gut microbiota profile and the accompanied host metabolism and physiology [11–14]. Meanwhile, compelling evidences have indicated that the gut microbiota might impact lipid metabolism in blood and tissue of the body via metabolites (e.g., short-chain fatty acids, trimethylamine, secondary bile acids, indole and its derivatives) and the bacterially proinflammatory factors (e.g., extracellular vesicles, peptidoglycan, DNA, lipopolysaccharide) [15–17]. However, the definitive and detailed associations among MAFLD, gut microbiota and lipid metabolism are still woefully inadequate [14, 18].

In this study, we took advantage of the human feces 16S amplicon technology and serum targeted quantification of lipid metabolism in combination with multiple clinical parameters to explore the multifaceted characteristics and candidate diagnostic biomarkers for dissecting patients with MAFLD and/or T2DM from health crowd. Collectively, our study suggested the similarities and distinctions in the clinical indicators, microbiome and metabolites, which would supply new references for the diagnosis and pathogenesis of MAFLD in future.

## Methods and materials

### Participants and specimen collection

During October and December of 2020, MAFLD and/or T2DM patients in Department of Gastroenterology and healthy volunteers in Physical Examination Center of the First Affiliated Hospital of Kunming Medical University were screened and grouped according to the inclusion and exclusion criteria as well as Declaration of Helsinki and the approval of the Ethics Committee of the First Affiliated Hospital of Kunming Medical University (Approval number: 2020-L-08; Approval date: January 20th, 2020). Informed consent was obtained from all participants (Ctr, MD and M) included in the study.

As to blood sample, 3 ml peripheral blood were collected with anticoagulant tubes containing EDTA as we recently reported. The serum should be isolated from blood samples within 30 min and stored in − 80 °C. As to microflora samples, over 500 mg faeces were collected with sterile centrifugal tubes and stored in − 80 °C as well. The detailed information of the participants was available in Additional file 3: Additional Table S1, and Additional file 4: Additional Table S2.

### The inclusion and exclusion criteria

As mentioned above, among the 200 patients (100 M patients, 100 MD patients) and 100 healthy candidates, 20 patients with M or MD and 20 Ctr were enrolled in the study. Generally, the inclusion and exclusion criteria for MAFLD were according to the international expert consensus statement [4]. For instance, the criteria for MAFLD were based on the evidence of hepatic steatosis, and one of the following three criteria, including overweight/obesity, evidence of metabolic dysregulation, or presence of type 2 diabetes mellitus [4]. In details, the inclusion criteria were mainly based on the controlled attenuation parameter (CAP) value and liver stiffness measurement (LSM) value measured by fibroscan (502 model, Echosens, France). The M and MD patients with a CAP value over 238 dB/m, while the Ctr with that lower than 238 dB/m instead. Additionally, the participants should stop using motility drugs and laxatives at least 1 week prior to breath test.

The exclusion criteria were: (1) an alcohol consumption history, over 140 g/week for men and over 70 g/week for women; (2) other diseases that can lead to fatty liver, including viral hepatitis, drug-induced liver disease, total parenteral nutrition, Wilson’s disease and autoimmune liver disease; (3) Age < 45, pacemaker installed, unhealed wounds in right upper abdomen and ascites; (4) malignancy or other terminal diseases including serious liver, kidney, heart, brain diseases and malignant tumors; (5) Subjects who have taken antibiotics in the last 4 weeks, or those have received clean enema and colonoscopy in the last 2 weeks.

### Microbiome and metabolite analyses

Faeces and peripheral blood samples were collected from the indicated groups (Ctr, MD, M), and then turned to 16S amplicon technology-based sequencing and gas/liquid chromatography-based serum targeted quantification of lipid metabolism for microbiome and metabolite analyses, respectively. The RNAs were sent to quality test and Novogene (Tianjin, China) for sequencing. The bioinformatic analyses of the data were completed by utilizing the software and platforms including Venn Map diagram, HeatMap, Principal Component Analysis (PCA) as we previously described [19–21].

### Statistical analysis

All statistical analysis was performed as we recently reported [21–23]. The GraphPad Prism 6.0 (GraphPad Software, USA) software was adopted for statistical analysis. The data were shown as mean ± SD (*N* = 3 independent experiments) and the One-way ANOVA analyses were used for the comparison of the indicated groups. Only when *P* < 0.05 was considered statistically significant. NS, not significant; *, *P* < 0.05; **, *P* < 0.01; ***, *P* < 0.001.

## Results

### FBG value revealed preferable correlation with MAFLD and/or T2DM patients

**Fig. 1:**
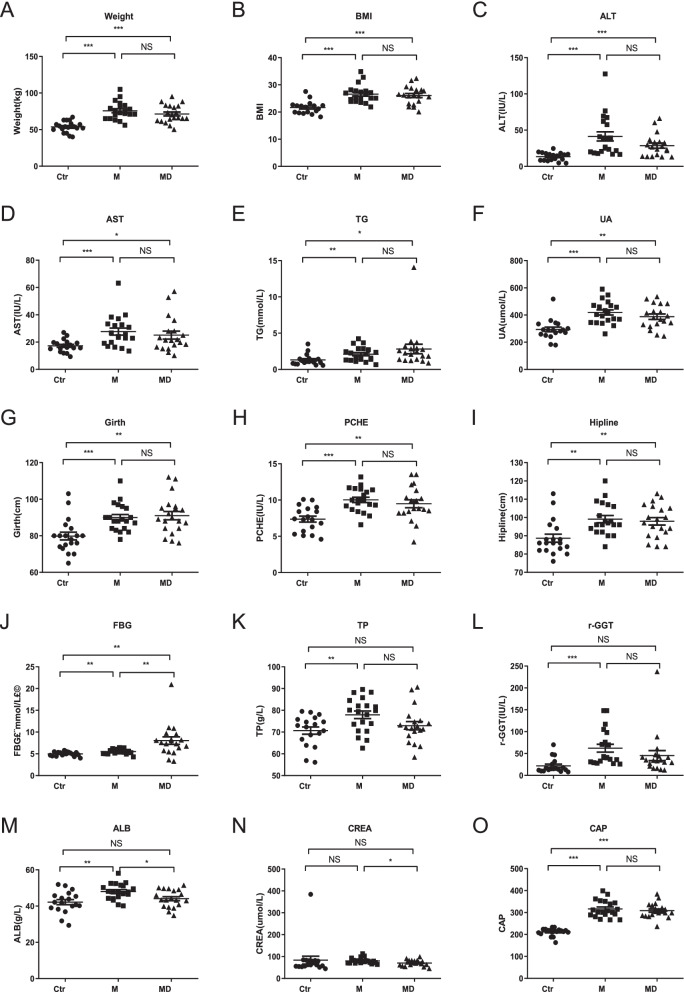
The clinicopathological indicators involved in M and MD patients and Ctr. **A-M** The comparations of clinicopathological parameters among Ctr, M and MD, including Weight (**A**), BMI (**B**), ALT (**C**), AST (**D**), TG (**E**), UA (**F**), Girth (**G**), PCHE (**H**), Hipline (**I**), FBG (**J**), TP (**K**), r-GGT (**L**), ALB (**M**), CREA (**N**), and CAP (**O**). All data are shown as Mean ± SEM. *, *P* < 0.05; **, *P* < 0.01; ***, *P* < 0.001; NS, not significant

From October to December, 2020, a total number of 40 patients (20 M, 20 MD) and 19 healthy participants (Ctr) were enrolled from the 300 candidates including 100 M, 100 MD and 100 Ctr according to the inclusion and exclusion criteria (Additional file 3: Additional Table S1, Additional file 4: Additional Table S2). As shown by the statistical analyses, patients with M or MD revealed distinctions in multiple clinical parameters compared to those in the Ctr group, including body weight, body mass index (BMI), alanine aminotransferase (ALT), aspartate aminotransferase (AST), triglyceride (TG), uric acid (UA), Girth, pseudocholinesterase (PCHE), Hipline, fasting blood glucose (FBG), while no significant differences between the M and MD groups were observed in the aforementioned indicators except FBG (Fig. 1A-J, Additional file 2: Additional Fig. S1A-S1F, Additional file 1: Additional Information, Additional file 3: Additional Table S1).

Compared to the Ctr group, the values of total protein (TP), γ-Glutamyl transferase (r-GGT) and albumin (ALB) in M patients rather than those in the MD cases exhibited statistical differences (Fig. 1K-M, Additional file 3: Additional Table S1). Furthermore, we also found a certain number of clinical parameters showed distinctions between M and MD patients such as FBG, ALB and creatinine (CREA) (Fig. 1J, M-N). As to other indicators involved in liver disease (e.g., HDL, ALP, BUN) and tumor (AFP, CA199, CEA) diagnosis, minimal differences were observed among the indicated three groups (Additional file 2: Additional Fig. S1B-S1N, Additional file 1: Additional Information). Notably, the controlled attenuation parameter (CAP) value between the Ctr and M, or Ctr and MD groups showed significant differences, which was consistent with our recent reports (Fig. 1O) [2]. Collectively, we verified that only the noninvasive FBG distribution basically satisfied the distinction of patients with MAFLD and/or T2DM from healthy participants.

### Patients with MAFLD and T2DM manifested distinguishable profiles of microbiota

**Fig. 2:**
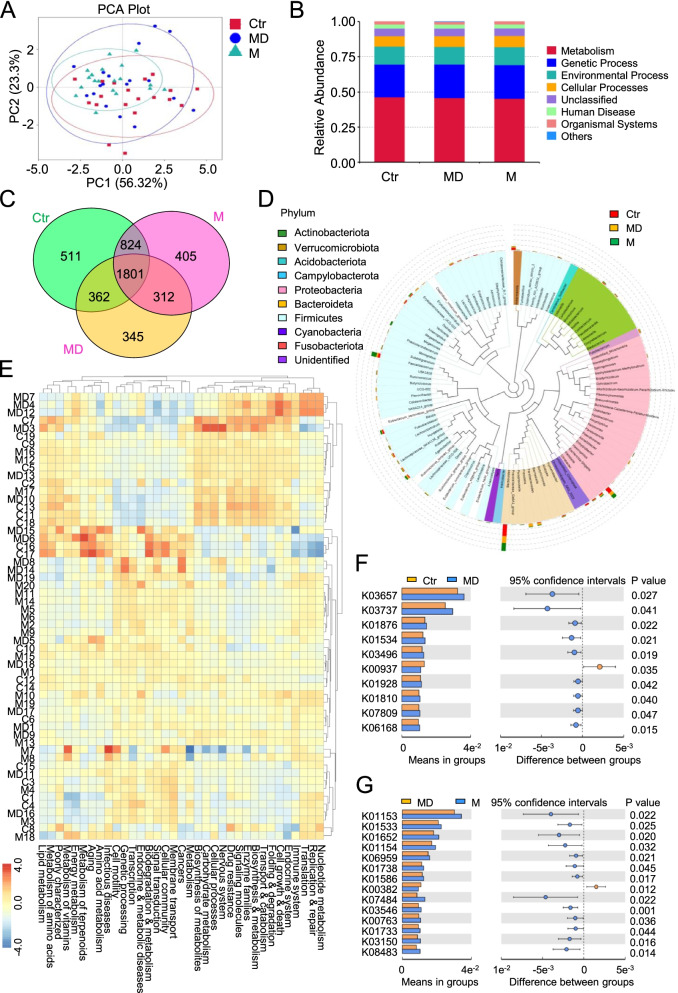
The comparation of microbiota profiles among the indicated groups. **A** The PCA (principal component analysis) of microbiota among the indicated groups (Ctr, M, MD) based on PC1 and PC2 values. **B** Histograms of microbiota-associated biological processes in the indicated groups. **C** Venn Map analysis of the distributions of the distribution of microbiota in stool samples among the indicated groups. **D** Krona genus evolutionary tree showed the phylum of the enriched microflora. **E** Hierarchical cluster analysis showed the correlation of the participants with the enriched biofunctions in the indicated groups (Ctr, M and MD). **F-G** Kyoto encyclopedia of genes and genomes (KEGG) showed the significant differences between Ctr and MD, or between MD and M

Having dissected the clinical relationship of multiple parameters with MAFLD and/or T2DM patients, we next turned to microbiome analysis for further exploring the candidate indicators by utilizing 16S amplicon technology-based sequencing. Generally, we found that the microbiomes in the indicated three groups revealed unique spectrum in spots of distribution and relative abundance as shown by the plots of principal components analysis (PCA) diagrams and species accumulation maps, respectively (Fig. 2A-B). Furthermore, the Venn Map and phylum diagrams intuitively exhibited the distribution of the 4560 kinds of common or distinct microbes among the Ctr, M and MD groups (Fig. 2C-D). For instance, a total of 1801 kind of gut microflora were reflected in the three groups, while only 511, 405 and 345 microflora were specifically enriched in the corresponding Ctr, M and MD groups, respectively (Fig. 2C). Interestingly, from the view of Krona genus evolutionary tree, we noticed that Bacteroideta, Proteobacteria and Firmicutes were the top 3 enriched microflora over those in the rest of phylum (Fig. 2D).

Hierarchical cluster analysis further exhibited the detailed correlation of the participants (Ctr, M and MD) with the enriched biofunctions in the indicated groups such as nucleotide metabolism, metabolism of terpenoids, folding and degradation (Fig. 2E). Additionally, within the 95% confidence intervals, a certain number of signaling pathways enriched by Kyoto encyclopedia of genes and genomes (KEGG) revealed significant differences between the enrolled healthy participants and MAFLD and/or T2DM patients (*P* < 0.05) (Fig. 2F-G).

### Patients with MAFLD and T2DM showed multifaceted diversity of microbiota

**Fig. 3:**
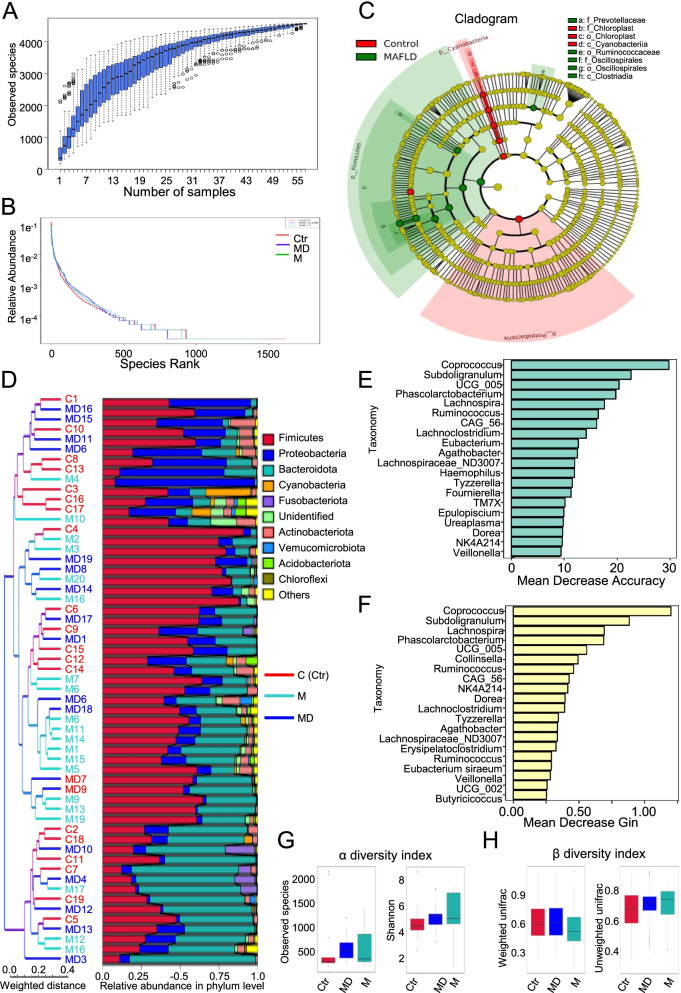
The multifaceted diversity of microbiota among the indicated groups. **A** Species accumulation boxplot showed the dynamic changes of species according to the number of samples. **B** The rank abundance curve showed the species richness and evenness of bacteria community among the indicated groups (Ctr, M, MD). **C** The cladogram of gut microflora among the indicated groups. **D** UPGMA (Unweighted Pair-group Method with Arithmetic Mean) analysis revealed the microbial communities and the relative species abundance at phylum level. **E** Random Forest analysis showed the taxonomy of microflora based on mean decrease accuracy (**E**) and mean decrease gin (**F**), respectively. **G-H** α diversity (**G**) and β (**H**) diversity index analyses revealed the between-group variance of species among the indicated groups

After evaluating the overview of the profile, we next explored the detailed similarities and differences of microbiota in the aforementioned groups. As shown by the species accumulation boxplot, we intuitively observed the sharp rise of species in the range of 1 and 19, whereas limited increase in species in the range of 28 and 55 (Fig. 3A). According to the rank abundance curve, the species richness and evenness of bacteria community revealed minimal variations among the three groups (Fig. 3B). Simultaneously, with the aid of the LEfSe (LDA effect size) methods, we conducted analysis of different species between groups and identified a series of biomarkers as shown by the cladogram (Fig. 3C). For example, the cladogram of gut microflora with significant differences between the Ctr (Control) and M (MAFLD patients) groups were exhibited such as Prevotellaceae, Chloroplast, Cyanobacteriia, Ruminococcaceae, Oscillospirales and Clostriadia (Fig. 3C).

Therewith, we took advantage of the weighted unifrac distance matrix for the UPGMA (Unweighted Pair-group Method with Arithmetic Mean) analysis to explore the composition of microbial communities and the relative species abundance of each sample at phylum level in the indicated samples. Interestingly, microbiota within and between the aforementioned groups collectively manifested significant diversity in phylum, which suggested the complexity of bacteria the community and species among the individuals (Fig. 3D). Furthermore, by performing the random forest analysis, we respectively analyzed the top 20 species abundance, and verified that Coprococcus, Subdoligranulum and UCG_005 were the top 3 species based on the analysis of mean decrease accuracy, while Coprococcus, Subdoligranulum and Lachnospira were the top 3 species based on the analysis of mean decrease gin (Fig. 3E-F). Finally, with the aid of α and β diversity index analyses, we found that the between-group variance of species among the indicated groups showed moderate differences (Fig. 3G-H).

### The diversity of the characteristics and correlations of 16S-based microbiota and lipid metabolism

**Fig. 4:**
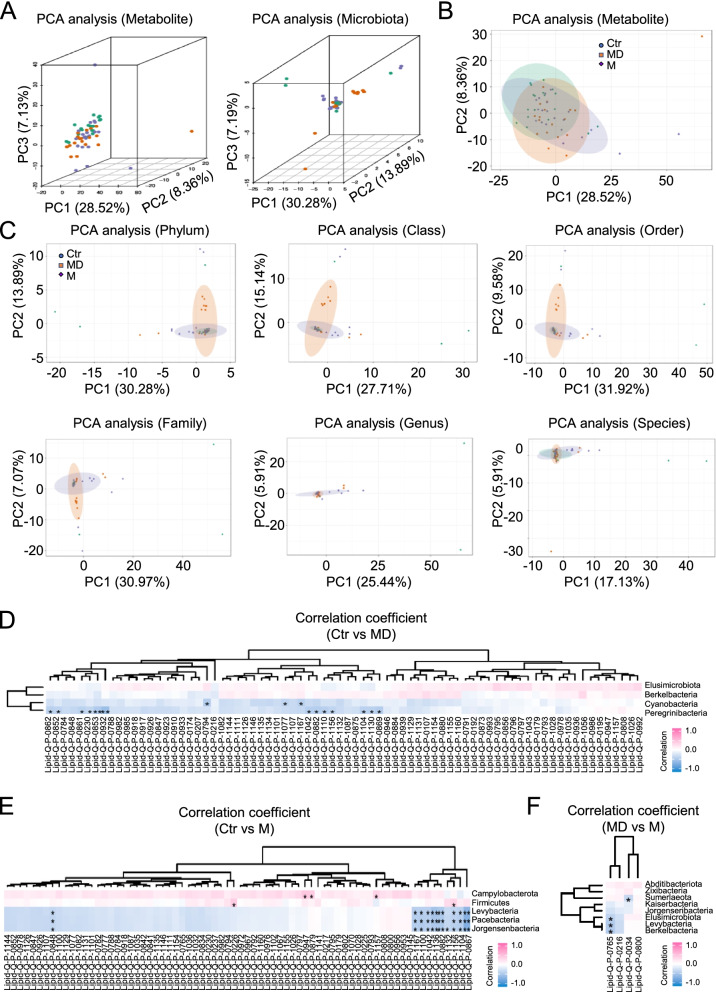
The overview of characteristics and correlations of microbiota and lipid metabolism. **A** PCA diagrams of metabolite and microbiota in the stool samples based on PC1, PC2 and PC3 values among the indicated groups (Ctr, M, MD). **B** PCA diagrams of metabolite in the stool samples based on PC1 and PC2 values among the indicated groups. **C** PCA diagrams of lipid metabolism on a series of levels including phylum, class, order, family, genus and species. **D-F** Sperman correlation-based hierarchical clustering analysis revealed the diversity in gut microbiota and lipid metabolism between Ctr and MD (**D**), Ctr and M (**E**), MD and M (**F**). *, *P* < 0.05

To explore the correlation of microbiome and metabolite in the indicated groups, we further turned to 16S-based gut microbiota and lipid metabolism analyses. Compared to the microbiota distribution, all of the aforementioned groups (Ctr, M, MD) revealed distinguishable pattern of metabolite according to the PCA diagrams (Fig. 4A-B). Interestingly, the PC1 and PC2 based PCA analysis further reflected the detailed similarities and differences of lipid metabolism among the individuals of the enrolled participants on a series of levels including phylum, class, order, family, genus and species (Fig. 4C). Intuitively, as shown by the Sperman correlation-based hierarchical clustering analysis, the significantly different microflora among the three groups revealed diversity in the pattern of gut microbiota and lipid metabolism (Fig. 4D-F). For example, the Peregrinibacteria and representative lipid metabolite (e.g., Lipid-Q-P-0862, Lipid-Q-P-0794, Lipid-Q-P-0869) in the MD group showed significantly negative correlation coefficient compared to the Ctr group (*P* < 0.05), while Firmicutes and Lipid-Q-P-1156 between the Ctr and M groups and Sumerlaeota and Lipid-Q-P-0034 between the MD and M groups revealed positive and negative correlation coefficient, respectively (Fig. 4D-F).

**Fig. 5:**
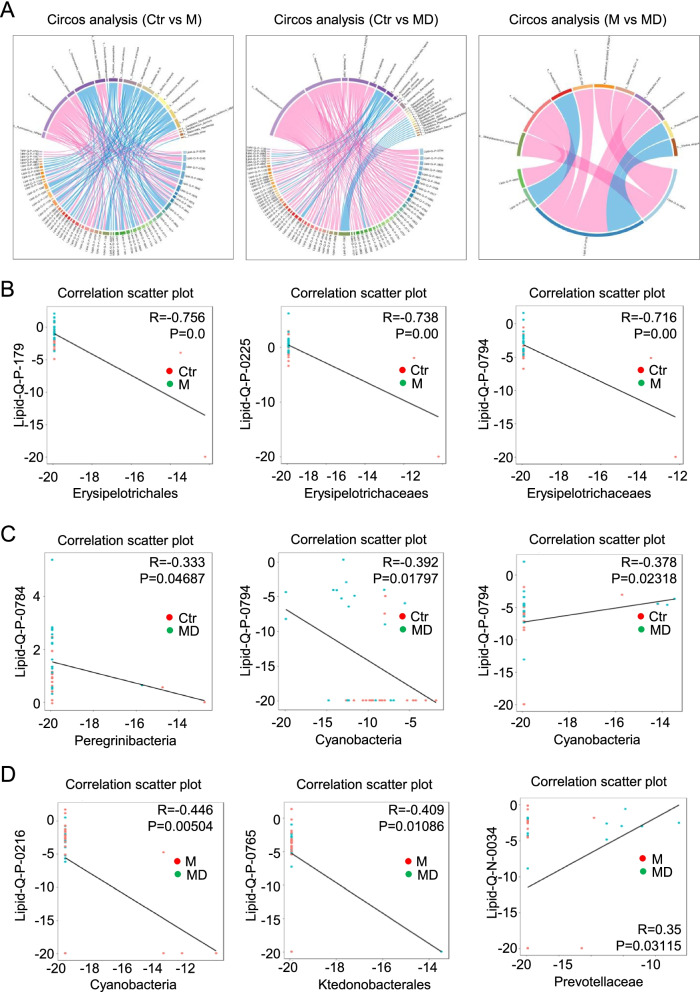
The detailed correlations of microbiota and lipid metabolism among the indicated groups. **A** Circos diagrams showed the correlation between the species of gut microbiome and metabolite in the indicated groups (Ctr, M, MD). **B-D** Correlation scatter plots showed the correlation of representative microbiome and metabolite between Ctr and MD (**B**), Ctr and M (**C**), MD and M (**D**)

Furthermore, the spatial correlation between the species of the indicated gut microbiome and metabolite was intuitive presented by the Circos diagrams among the Ctr, M and MD groups (Fig. 5A). Furthermore, the representative microbiome and metabolite with significant positive or negative correlations (*P* < 0.05, |r| ≥ 0.3) between the indicated groups were shown by the correlation scatter plots as well (Fig. 5B-D). For instance, Erysipelotrichaceaes had negative correlation with multiple lipid metabolism (e.g., Lipid-Q-P-179, Lipid-Q-P-0225, Lipid-Q-P-0794) between the Ctr and M groups, whereas Lipid-Q-P-0794 showed negative and positive correlations with Cyanobacteria and Cyanobacteria between the Ctr and MD groups, respectively (Fig. 5B-C). As to the M and MD groups, we also observed the diversity of the correlations between the representative microbiome (e.g., Cyanobacteria, Ktedonobacterales, Prevotellaceae) and the corresponding metabolite (e.g., Lipid-Q-P-0216, Lipid-Q-P-0765, Lipid-Q-N-0034) (Fig. 5D).

## Discussion

The pathophysiology underlying MAFLD is a long-lasting issue, which comprises a multitude of interlinked processes such as insulin resistance, lipotoxicity, infiltration of proinflammatory cells, hepatic stellate cell (HSC) fibrogenesis and over-activation [24, 25]. Despite the aforementioned well-established processes, yet the molecular mechanisms are incompletely uncovered and the approved pharmacological treatments for MAFLD are also unavailable [1, 26, 27]. For the purpose of further verifying the detailed pathogenesis of patients with MAFLD and/or T2DM for developing novel treatment remedies, we took advantage of the 16S-based sequencing and serum targeted quantification of lipid metabolism for gut microbiome and metabolite analyses. Notably, we characterized the similarities and diversities of the microbiome and metabolite among health participants, MAFLD and/or T2DM patients, and verified the correlation of representative microbiome and the corresponding metabolite that held promising prospect as novel biomarkers and therapeutic targets in dissecting and resolving the MAFLD-associated pathophysiology.

Current studies have put forward novel concepts underlying pathophysiology of MAFLD [27, 28]. On the one hand, the excess extracellular matrix (ECM) and proinflammatory cytokines secreted by activated HSCs, proinflammatory cells and damaged hepatocytes have been demonstrated to play a critical role in impairing the physiological structure and biofunctions of the liver [24, 26, 29]. On the other hand, recent advances in microbiome and metabolite have also indicated the involvement of dysfunctional gut microbiome, dysregulated lipid metabolism, intestinal fructose handling and endogenous alcohol production, together with abnormal regulation and alterations of multiple signaling cascades (e.g., Hedgehog, BMP, TGF-β) for modifying the individual susceptibility to MAFLD patients with T2DM [24]. Additionally, the obesity as well as T2DM has been involved with MAFLD and microbiota from pathophysiology to therapeutics as well [30, 31]. Consistently, our study revealed the multifaceted similarities and diversities of MAFLD patients with T2DM from the perspectives of gut microbiome and metabolites as well as clinical indicators.

Emerging evidences have suggested the metabolic disorders with various alterations in the composition of intestinal microbiota and relevant metabolites, which are adequate to translocate from the gut across to liver and thus contribute to metabolic liver disorders (e.g., MAFLD) or T2DM [14, 32, 33]. Patients with T2DM manifested features of insulin resistance including beta cell deterioration, augmented endogenous glucose production and decreased peripheral glucose uptake, which has been considered an integral pathogenesis for MAFLD development [34, 35].

The gut-liver axis is the consequence of bidirectional regulation between the anatomical structure of gut and liver, which thus determines the pivotal modulating effects of gut microbiota and the concomitant microbiome upon liver activity [36, 37]. To date, microbiome has been indicated with the involvement of the pathogenesis of MAFLD or obesity via generating harmful metabolites (e.g., short-chain fatty acids, secondary bile acids, branched amino acids, and indoles) [38, 39]. Very recently, Cerreto and their colleagues proposed the characteristics of the gut-liver axis in liver disorders, and Fianchi et al further confirmed the gut-liver interaction in the pathophysiology of MAFLD and the potential target for personalized treatment [40–42]. Furthermore, in this study, we conducted the gut microbiota and lipid metabolite analyses, and suggested the multifaceted characteristics and inherent correlations with pathophysiologic abnormalities among the healthy population and those patients with MAFLD and/or T2DM, which collectively supply new references for further exploring the gut microbiota-mediated pathogenesis as well as gut microbiome- or metabolite-targeted therapies on NAFLD in future.

## Conclusion

Gut microbiota and lipid metabolites in MAFLD and/or T2DM hold promising prospective for developing more accurate diagnosis and novel pharmacological treatments. Our findings based on microbiota and metabolite analyses would provide overwhelming new references for understanding the pathogenesis, diagnosis as we as facilitating the development of gut microbiome- or metabolite-based biomarkers and targeted therapies on MAFLD.

## Supplementary Information

**Additional file 1: Additional Information.** The detailed information accompanied with the main manuscript, including Additional Information (including Additional Figure Legend for Fig. S1).

**Additional file 2: Additional Figure S1.** The clinicopathological indicators involved in M and MD patients and Ctr.

**Additional file 3: Additional Table S1.** Characteristics of patients with MAFLD, MAFLD/T2DM.

**Additional file 4: Additional Table S2.** Clinical Parameters in M and MD patients and Ctr healthy participants.

## Data Availability

All data during this study are included in the published article. Meanwhile, the datasets analyzed during the study are available from the corresponding author upon reasonable request.

## Abbreviations

MAFLD: Metabolic associated fatty liver disease
T2DM: Type II diabetes mellitus
NAFLD: Non-alcoholic fatty liver disease
NASH: Nonalcoholic steatohepatitis
NAFL: Nonalcoholic fatty liver
GGT: γ-glutamyl transferase
HCC: Hepatocellular carcinoma
CAP: Controlled attenuation parameter
LSM: Liver stiffness measurement
PCA: Principal Component Analysis
BMI: Body mass index
ALP: Alkaline phosphatase
ALT: Alanine aminotransferase
AST: Aspartate aminotransferase
FBG: Fasting blood glucose
TG: Triglyceride
UA: Uric acid
HSC: Hepatic stellate cell
ECM: Extracellular matrix
BUN: Blood urea nitrogen
TB: Total bilirubin
HDL-C: High-density lipoprotein-cholesterol
LDL-C: Low-density lipoprotein-cholesterol
CREA: Creatinine
TC: Total cholesterol
PChE: Pseudocholinesterase
PLT: Platelet count
