# Supplementary material for: Characterization of microbiome and metabolite analyses in patients with metabolic associated fatty liver disease and type II diabetes mellitus
Source: BMC Microbiol. 2022 Apr 15;22:105. doi: 10.1186/s12866-022-02526-w (PMC9011963; doi:10.1186/s12866-022-02526-w)
Supplement: Supplementary file 1 — Additional file 1: Additional Information. The detailed information accompanied with the main manuscript, including Additional Information (including Additional Figure Legend for Fig. S1). [file 12866_2022_2526_MOESM1_ESM.docx]

**Additional Information**

**Characterization of Microbiome and Metabolite Analyses in Patients with Metabolic Associated Fatty Liver Disease and Type Ⅱ Diabetes Mellitus**

Qiuping Yang^1^**^§^**, Leisheng Zhang^2,3^**^§^**^*^, Qian Li^4^**^§^**, Man Gu^1^, Qiu Qu^1^, Xinglong Yang^1^, Qinghua Yi^5^, Kunli Gu^1^, Linli Kuang ^1^, Mei Hao^1^, Jing Xu^1^, Hongju Yang^1*^

^1^ Division of Gastroenterology, The First Affiliated Hospital of Kunming Medical University, Kunming, 650031, China

^2^ The National Postdoctoral Research Station, Gansu Provincial Hospital, Lanzhou, 730013, China

^3^ Key Laboratory of Radiation Technology and Biophysics, Institute of Biology & Hefei Institute of Physical Science, Chinese Academy of Sciences, Hefei, 230031, China

^4^ Transfusion Medicine Research Department, Yunnan Kunming Blood Center, Kunming, 650011, China

^5^ Kunming Guandu District People's Hospital, Kunming, 650220, China

**Additional Information**

**Additional Figure Legends**

**Additional Figure Legends**

**Figure S1. The clinicopathological indicators involved in M and MD patients and Ctr.** (**A-N**) The comparations of age distribution (**A**), clinicopathological parameters and tumor-associated indicators among Ctr, M and MD, including HDL (**B**), ALP (**C**), BUN (**D**), LDL (**E**), TC (**F**), TBIL (**G**), Fibroscan (E) (**H**), AFP (**I**), CA125 (**J**), CA153 (**K**), CA199 (**L**), CA724 (**M**) and CEA (**N**). All data are shown as Mean ± SD. *, *P*<0.05; NS, not significant.
