# Supplementary figures and images for: Characterization of microbiome and metabolite analyses in patients with metabolic associated fatty liver disease and type II diabetes mellitus
Source: BMC Microbiol. 2022 Apr 15;22:105. doi: 10.1186/s12866-022-02526-w (PMC9011963; doi:10.1186/s12866-022-02526-w)

A

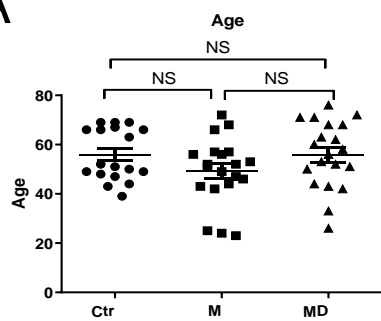

B

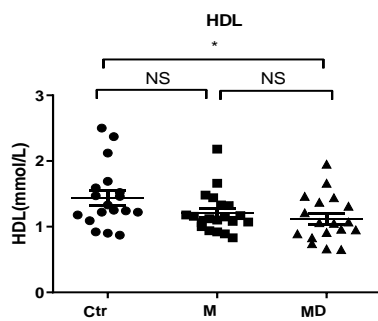

C

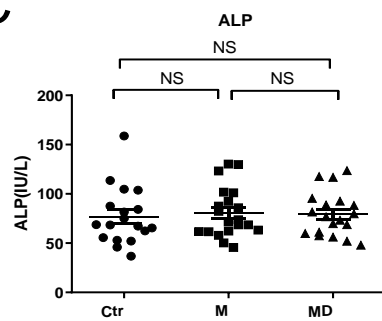

D

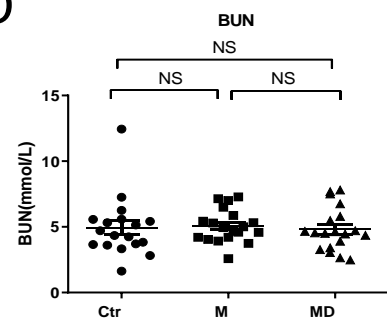

E

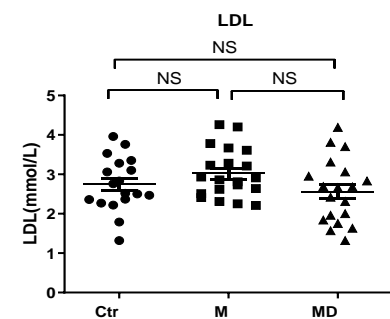

F

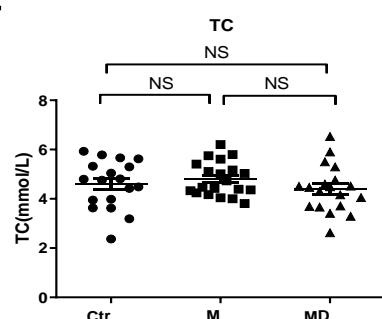

G

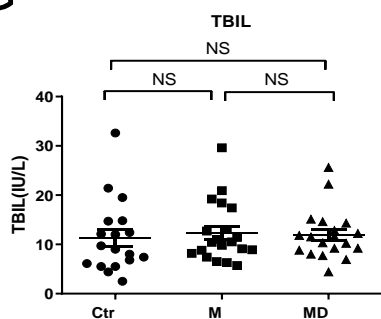

H

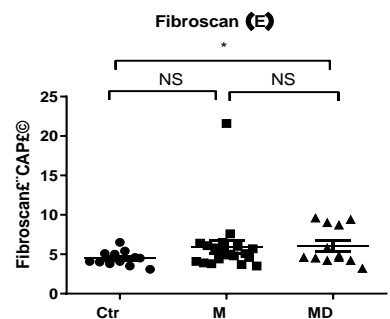

I

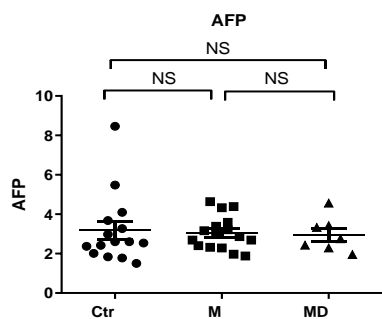

J

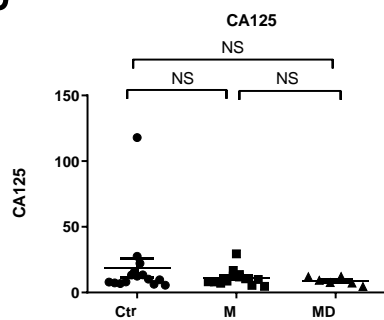

K

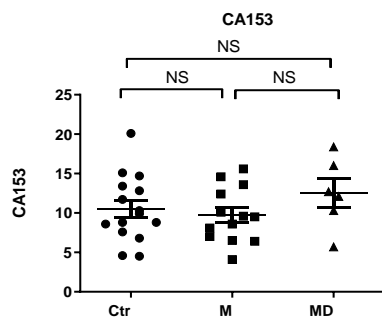

L

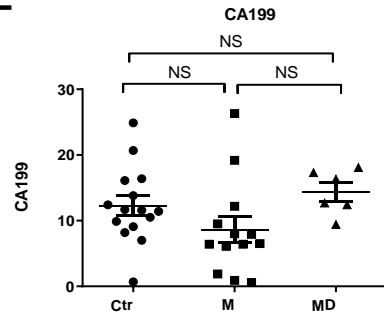

M

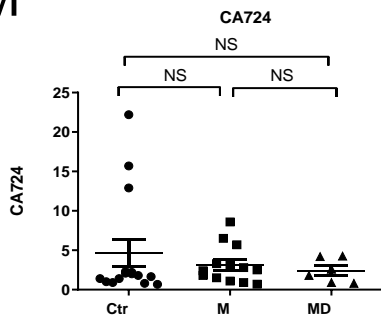

N

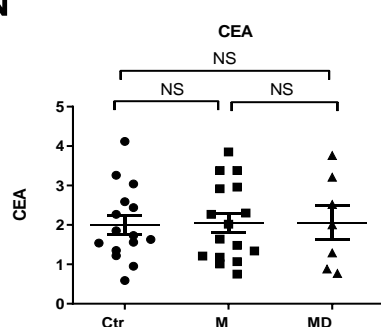

Supplement: Supplementary file 2 — Additional file 2: Additional Figure S1. The clinicopathological indicators involved in M and MD patients and Ctr. [file 12866_2022_2526_MOESM2_ESM.pdf]
