# Supplementary material for: Characterization of microbiome and metabolite analyses in patients with metabolic associated fatty liver disease and type II diabetes mellitus
Source: BMC Microbiol. 2022 Apr 15;22:105. doi: 10.1186/s12866-022-02526-w (PMC9011963; doi:10.1186/s12866-022-02526-w)
Supplement: Supplementary file 3 — Additional file 3: Additional Table S1. Characteristics of patients with MAFLD, MAFLD/T2DM. [file 12866_2022_2526_MOESM3_ESM.docx]

**Additional Table S1** **Characteristics of patients with MAFLD, MAFLD/T2DM**

| Parameters | Control (n=19) | M (n=20) | MD (n=20) | Total (n=59) | P value |
| --- | --- | --- | --- | --- | --- |
| Gender (male/female) | 7/12 | 14/6 | 13/7 | 34/25 | 0.1674 |
| Age | 55.37±2.43 | 49.15 ±3.01 | 55.95±3.05 | 53.46±1.67 | 0.1812 |
| Height | 1.58±0.02 | 10.00±8.32 | 1.65±0.02 | 4.46±2.82 | 0.3780 |
| Weight | 53.68±1.71 | 75.70±2.68 | 71.35 ±2.72 | 67.14±1.86 | < 0.0001 |
| Girth | 79.84 ±2.13 | 89.95±1.69 | 91.00 ±2.29 | 87.70 ±1.36 | 0.0005 |
| Hipline | 88.68±2.19 | 99.10 ±1.96 | 97.90 ±2.01 | 95.34 ±1.31 | 0.0012 |
| Waist hip ratio | 0.90±0.01 | 0.8950 ±0.01 | 0.92±0.01 | 0.91±0.004 | 0.0508 |
| BMI | 21.39±0.41 | 26.60±0.73 | 25.99±0.72 | 24.71±0.47 | < 0.0001 |
| Hypertension (no/yes) | 14/5 | 11/9 | 12/8 | 37/22 | 0.6708 |
| coronary heart disease (no/yes) | 17/2 | 18/2 | 16/4 | 51/8 | 0.7836 |
| atherosclerosis (no/yes) | 16/3 | 17/3 | 10/10 | 43/16 | 0.0456 |
| diabetes (no/yes) | 19/0 | 19/1 | 3/17 | 41/18 | < 0.0001 |
| abdominal pain (no/yes) | 7/12 | 13/7 | 14/6 | 34/25 | 0.1674 |
| abdominal distension (no/yes) | 11/8 | 13/7 | 15/5 | 39/20 | 0.7318 |
| diarrhea (no/yes) | 13/6 | 17/3 | 16/4 | 46/13 | 0.6522 |
| constipation (no/yes) | 16/3 | 18/2 | 17/3 | 51/8 | 0.9539 |
| fatty liver (no/slight/ moderate/gravity) | 19/0/0/0 | 0/0/4/16 | 1/8/0/11 | 20/8/4/27 | < 0.0001 |
| Autoimmune liver (no/PBC/PBS/AIH/overlap) | 19/0/0/0/0 | 20/0/0/0/0 | 20/0/0/0/0 | 59/0/0/0/0 | --- |
| alcoholic liver (no/yes) | 19/0 | 20/0 | 20/0 | 59/0 | --- |
| viral hepatitis(no/Hepatitis a/hepatitis b/hepatitis c/others） | 19/0/0/0/0 | 20/0/0/0/0 | 20/0/0/0/0 | 59/0/0/0/0 | --- |
| hepatic sclerosis (no/yes) | 19/0 | 20/0 | 20/0 | 59/0 | --- |
| tumor (no/yes) | 19/0 | 20/0 | 20/0 | 59/0 | --- |
| TP | 70.67 ±1.57 | 77.93±1.74 | 72.96 ±1.78 | 73.91 ±1.05 | 0.0127 |
| ALB | 42.43 ±1.37 | 48.02 ±0.99 | 44.12 ±1.06 | 44.90 ±0.72 | 0.0035 |
| TBIL | 11.11±1.66 | 12.26 ±1.35 | 11.75±1.12 | 11.72 ±0.79 | 0.8413 |
| ALT | 13.24 ±1.29 | 41.33±6.32 | 28.42 ±3.39 | 27.90 ±2.86 | 0.0001 |
| AST | 17.28 ±1.02 | 27.67±2.58 | 24.92 ±2.80 | 23.39 ±1.43 | 0.0074 |
| ALP | 76.09 ±6.57 | 80.98 ±5.67 | 77.64 ±5.18 | 78.27 ±3.31 | 0.8319 |
| PCHE | 7.42 ±0.39 | 10.03 ±0.37 | 9.45 ±0.52 | 8.99 ±0.29 | 0.0002 |
| r-GGT | 21.37±3.80 | 62.22 ±9.02 | 45.82 ±10.85 | 43.50 ±5.33 | 0.0056 |
| Cr | 83.71 ±16.97 | 80.00±2.82 | 69.99 ±2.99 | 77.80 ±5.59 | 0.5924 |
| BUN | 4.98 ±0.51 | 5.07±0.29 | 4.91 ±0.37 | 4.98 ±0.23 | 0.9598 |
| UA | 300.2±17.69 | 418.8 ±18.67 | 385.4 ±19.03 | 369.3 ±12.35 | 0.0001 |
| TC | 4.61 ±0.22 | 4.80±0.15 | 4.40 ±0.21 | 4.60 ±0.11 | 0.3372 |
| TG | 1.31±0.17 | 2.10±0.22 | 2.74 ±0.63 | 2.06 ±0.24 | 0.0511 |
| HDL | 1.44 ±0.11 | 1.20±0.07 | 1.13 ±0.08 | 1.26 ±0.05 | 0.0370 |
| LDL | 2.77±0.15 | 3.02±0.14 | 2.55 ±0.18 | 2.780 ±0.09 | 0.1123 |
| FBG | 4.97 ±0.11 | 5.52 ±0.12 | 7.98±0.828 | 6.17 ±0.33 | 0.0001 |
| CAP | 211.5 ±3.85 | 316.5 ±8.62 | 308.7 ±7.34 | 280.0 ±7.37 | <0.0001 |
| E | 4.48±0.17 | 5.94±0.86 | 6.03 ±0.41 | 5.50±0.33 | 0.1104 |
| fatty liver (no/slight/moderate/gravity) | 19/0/0/0 | 0/0/5/15 | 1/1/3/15 | 20/1/8/30 | < 0.0001 |
| Fibrosis (F0<6.6kPa, no fibrosis/F1: 6.6-8.0kPa, mild fibrosis/F2: 8-11kPa, significant fibrosis/F3≥11.0kPa, the advanced fibrosis/F4≥15.0kPa, hepatic sclerosis) | 19/0/0/0/0 | 18/1/0/1/0 | 16/0/4/0/0 | 53/1/4/1/0 | --- |
